# Broncholith Removal Using a Cryoprobe in Combination With Biopsy Forceps in a Patient With Immune Thrombocytopenia: A Case Report

**DOI:** 10.1002/rcr2.70779

**Published:** 2026-10-21

**Authors:** Qisheng Xiao, Nan Ma, Jianbo Zhu

**Affiliations:** ^1^ Department of Respiratory and Critical Care Medicine Binzhou People's Hospital Affiliated to Shandong First Medical University Binzhou Shandong China

**Keywords:** broncholith, bronchoscopy, thrombocytopenia

## Abstract

Broncholithiasis is a rare respiratory condition usually caused by calcified lymph nodes eroding into the bronchial lumen, often following pulmonary tuberculosis or histoplasmosis infections. Patients may be asymptomatic or present with haemoptysis, recurrent respiratory infections or airway obstruction, for which bronchoscopic or surgical treatment may be required. No internationally standardized guidelines currently exist for broncholith removal. We report a 63‐year‐old woman with previous pulmonary tuberculosis, recurrent haemoptysis and thrombocytopenia that was subsequently diagnosed as immune thrombocytopenia. Chest CT showed a 9 × 8 mm calcified lesion in the distal anterior segmental bronchus of the right upper lobe. Bronchoscopy revealed a transbronchial broncholith surrounded by granulation tissue and firmly attached at its base. The broncholith was removed using a cryoprobe and biopsy forceps under flexible bronchoscopy, with minor bleeding. This case suggests that broncholith removal under flexible bronchoscopy using a cryoprobe combined with biopsy forceps may be feasible in patients with thrombocytopenia.

## Graphical abstract

A 63‐year‐old woman with a history of pulmonary tuberculosis had been hospitalized twice for haemoptysis. During her hospital stay, broncholith extraction was performed under flexible bronchoscopy using a cryoprobe in combination with biopsy forceps. The extracted broncholith fragmented during removal.

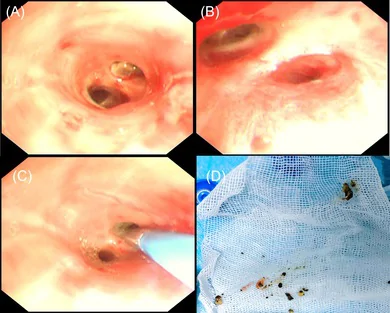

## 1 Introduction

Broncholithiasis is a relatively rare respiratory condition, and the broncholith are often caused by tuberculosis and histoplasmosis infections [1]. Some patients may be asymptomatic; however, when symptoms such as haemoptysis, recurrent infections or airway obstruction occur, the broncholith removal may be considered [1, 2]. Currently, there is no internationally agreed‐upon standardized approach to broncholith management. This case demonstrates that a cryoprobe combined with biopsy forceps under flexible bronchoscopy may be a feasible therapeutic option for selected patients with transbronchial broncholith, and that a platelet count below 50 × 10^9^/L is not a contraindication for broncholith removal.

## 2 Case Report

A 63‐year‐old woman was admitted to the hospital twice for haemoptysis; she had a history of pulmonary tuberculosis. She had no history of alcohol consumption or smoking. During her first hospitalization, laboratory investigations revealed a platelet count of 66 × 10^9^/L. A CT scan showed a calcified lesion in the distal anterior segmental bronchus of the right upper lobe. The patient was advised to determine the aetiology of her thrombocytopenia but refused; she was discharged after receiving conservative management.

She was readmitted for haemoptysis a second time, 6 months after her first admission. Laboratory investigations conducted during this hospitalization showed a platelet count of 56 × 10^9^/L. A contrast‐enhanced CT scan revealed a 9 × 8 mm calcified lesion in the distal anterior segmental bronchus of the right upper lobe, adjacent to several pulmonary vascular branches (Figure 1A,B). A preoperative platelet count was 49 × 10^9^/L. A multidisciplinary assessment was conducted in the hospital. After the patient signed the informed consent form, the broncholith was removed via flexible bronchoscopy under local anaesthesia. During the procedure, the broncholith was surrounded by granulation tissue and firmly anchored at its base. (Figure 2A). Because the broncholith is tightly adherent to the bronchial wall and surrounding granulation tissue, and is located near several branches of the pulmonary vessels, direct removal of the entire broncholith would increase the risk of bleeding. A 1.1 mm diameter disposable cryoprobe (ARCTIQUEFOCUS, Arcticous Biotech (Shanghai) Co. Ltd., Shanghai, China) using CO_2_ was applied to the exposed broncholith for 3–5 s, Because fragmentation remained difficult, freezing was extended to 5–10 s. The cryoprobe‐assisted fragmentation and forceps extraction are repeated 2–4 times, allowing most of the broncholith to be removed in pieces without forceful pulling. (Figure 2B–D). To minimize the risk of major bleeding, small residual fragments at the base were not forcibly removed. Only minor bleeding was observed throughout the procedure. Subsequent hematologic evaluation confirmed a diagnosis of immune thrombocytopenia. A follow‐up CT scan 1 month postoperatively showed that the calcified lesion had disappeared, and the patient had not experienced any further episodes of haemoptysis (Figure 1C).

**FIGURE 1:**
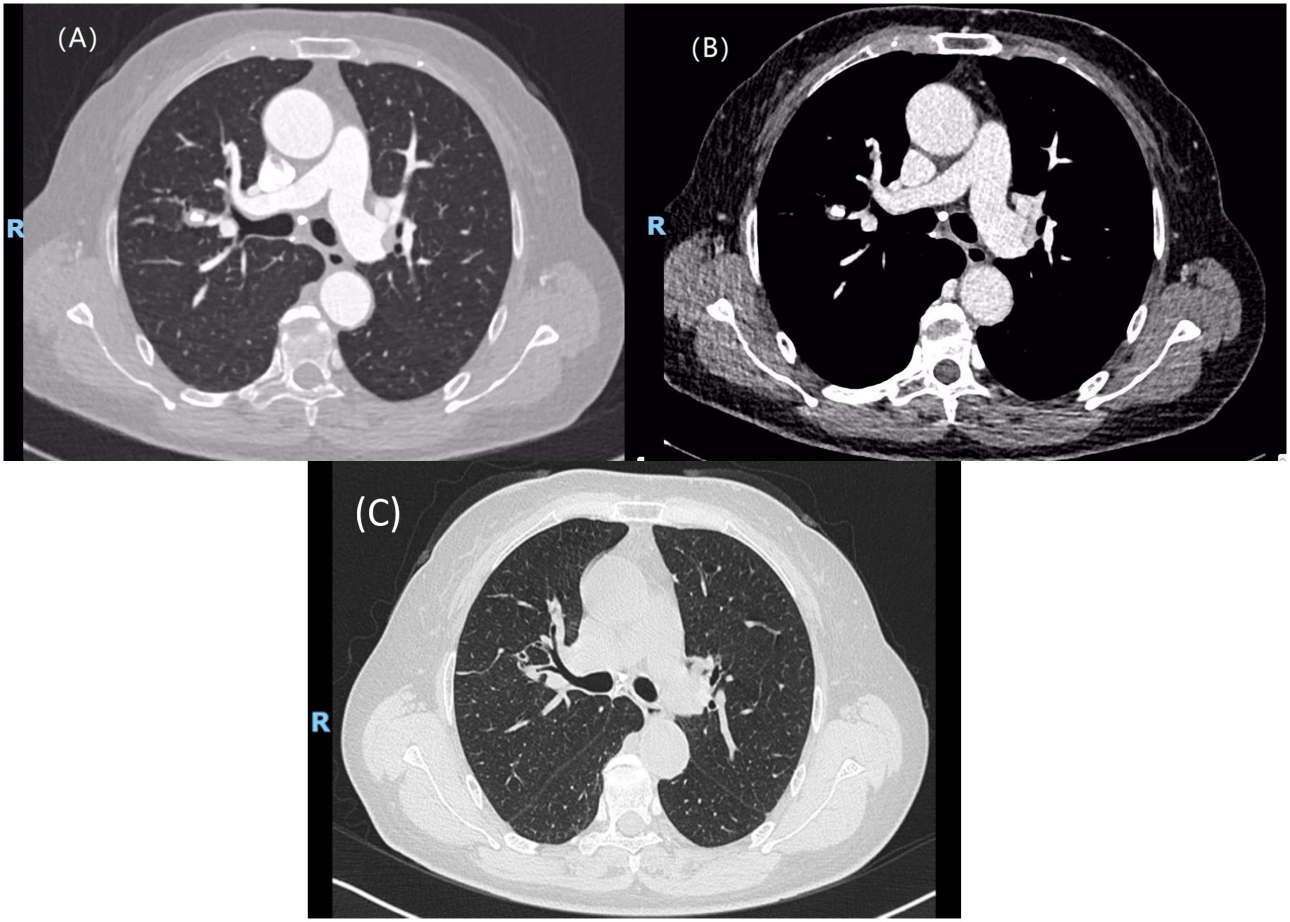
Chest computed tomography. (A) Lung window image showing a calcified lesion in the distal anterior segmental bronchus of the right upper lobe. (B) Mediastinal window image after intravenous contrast administration showing the broncholith. (C) Follow‐up chest CT obtained 1 month after bronchoscopic removal.

**FIGURE 2:**
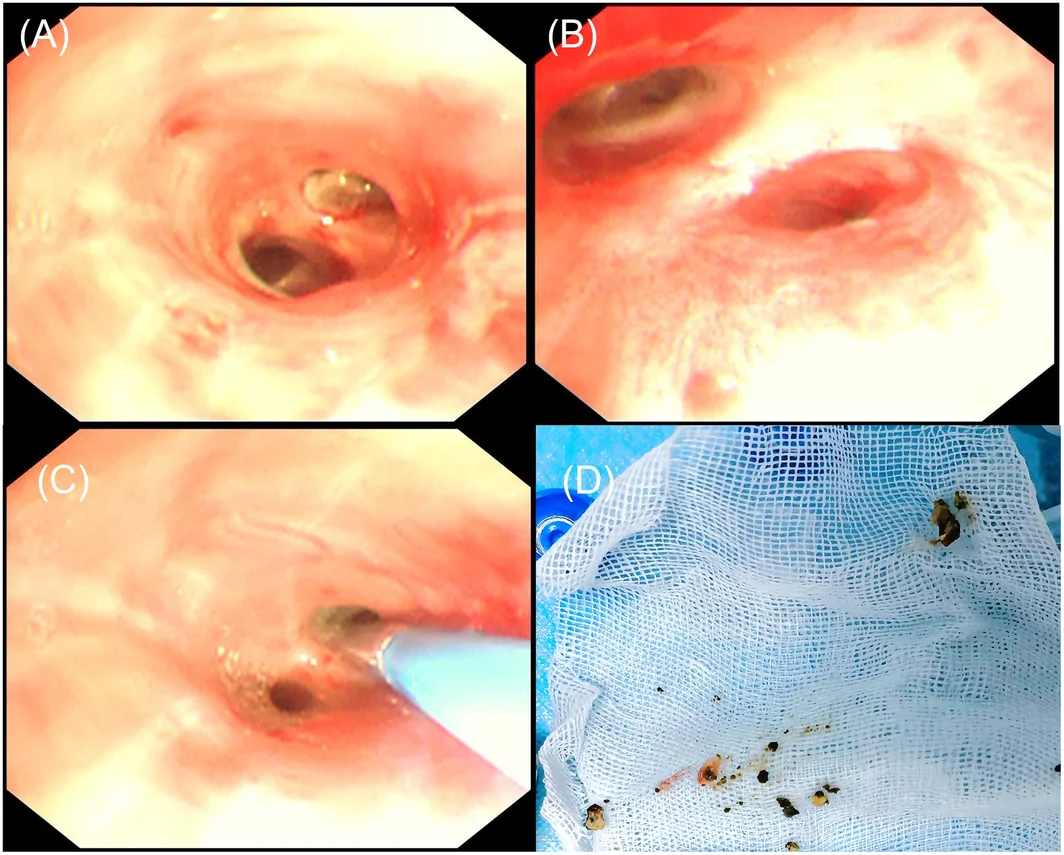
Bronchoscopic removal of the broncholith. (A) A broncholith surrounded by granulation tissue in the distal anterior segmental bronchus of the right upper lobe. (B) Bronchoscopic appearance after removal, with no visible residual broncholith. (C) Removal of the broncholith using a cryoprobe. (D) Extracted broncholith fragments.

## 3 Discussion

The clinical significance of this case is that for firmly attached distal broncholith, when forceful pulling carries a risk of bleeding, it may still be feasible to remove them in pieces using a cryoprobe with a flexible bronchoscope, even in the presence of thrombocytopenia.

Several methods have been described for broncholith extraction, including biopsy forceps, balloon catheters, laser treatment and cryotherapy [1, 2]. Previous studies have shown that cryotherapy may be useful for broncholith that are fixed or partially attached and difficult to remove with forceps [3, 4]. Cryotherapy may help to separate or fragment broncholith, thereby avoiding forcible traction when there is a high risk of bleeding [4]. Our case further suggests that this technique may be feasible in certain patients at high risk of bleeding, such as those with thrombocytopenia. Rigid bronchoscopy has been reported to have a higher success rate than flexible bronchoscopy [3, 5]. However, the broncholith in this patient was located in a distal bronchus. Finally, we selected flexible bronchoscopy using a cryoprobe combined with biopsy forceps. Preoperatively, performing contrast‐enhanced CT with reconstruction, conducting multidisciplinary assessment and preparing bronchial occlusion balloons are very important. Intraoperatively, cryotherapy should target the exposed broncholith, adjusting freezing time according to fragmentation difficulty, while avoiding strong traction.

Bleeding was the main concern during the procedure. The broncholith was firmly attached to the bronchial wall and surrounded by granulation tissue. Contrast‐enhanced CT also showed several pulmonary vascular branches close to the lesion. In addition, the preoperative platelet count was only 49 × 10^9^/L. After multidisciplinary discussion and preparation for possible bleeding, bronchoscopic extraction was performed.

The broncholith could not be removed using biopsy forceps alone. Repeated applications of the cryoprobe, together with gentle manipulation by biopsy forceps, gradually fragmented the broncholith. It was then removed piecemeal, without forceful traction. No further significant haemoptysis occurred after the procedure, and follow‐up CT showed disappearance of the previous calcified lesion.

Although this case cannot define a safe platelet threshold for broncholith removal, it suggests that broncholith removal under flexible bronchoscopy using a cryoprobe combined with biopsy forceps may be feasible in patients with thrombocytopenia. The main advantage of cryoprobes is their ability to facilitate broncholith fragmentation by freezing, allowing larger broncholiths to be removed piecemeal and reducing the need for forceful traction and the risk of bleeding [3, 4, 5]. This may provide an alternative to forceful en bloc extraction for firmly attached broncholiths or those with a high bleeding risk. Previous reports described cryoprobe‐assisted removal in the right middle lobe bronchus, right upper lobe bronchus and left upper lobe lingular segment bronchus [3, 4, 5]. In our case, the procedure was successfully performed in the fifth to seventh generation bronchi, suggesting that this technique may also be feasible in more distal bronchi. If the procedure is unsuitable under local anaesthesia, rigid bronchoscopy should be considered.

## Author Contributions

Qisheng Xiao collected the clinical data, reviewed the literature and drafted the manuscript. Nan Ma performed the bronchoscopic procedure, contributed to the interpretation of the clinical and imaging findings and revised the manuscript. Jianbo Zhu supervised the clinical management of the patient, contributed to the conception of the report and critically revised the manuscript.

## Funding

This work was supported by the Beijing Health Alliance Charitable Foundation (KM‐MXQD‐004).

## Consent

The authors declare that written informed consent was obtained for the publication of this manuscript and accompanying images using the consent form provided by the Journal.

## Conflicts of Interest

The authors declare no conflicts of interest.

## Data Availability

The data that support the findings of this study are available on request from the corresponding author. The data are not publicly available due to privacy or ethical restrictions.
